# Management of Hip Arthroplasty for Intertrochanteric Fractures Under the Treatment Concept of Periprosthetic Fractures

**DOI:** 10.1111/os.70164

**Published:** 2025-08-26

**Authors:** Wei‐Qiang Zhao, Xu‐Song Li, Ke‐Qin Yu, Rong‐Zhen Xie, Jiang Hua, Jie‐Feng Huang

**Affiliations:** ^1^ Department of Orthopaedics & Traumatology The First Affiliated Hospital of Zhejiang Chinese Medical University (Zhejiang Provincial Hospital of Chinese Medicine) Zhejiang Hangzhou China; ^2^ Zhejiang Chinese Medical University Zhejiang Hangzhou China; ^3^ Department of Orthopaedics and Traumatology Zhongshan Hospital of Traditional Chinese Medicine (Zhongshan Hospital of Traditional Chinese Medicine Affiliated to Guangzhou University of Traditional Chinese Medicine). Zhongshan Guangdong China

**Keywords:** dual‐cable fixation, elderly patients, hip arthroplasty, intertrochanteric fractures, long‐stem prosthesis, periprosthetic femoral fracture treatment concept

## Abstract

**Objective:**

Intertrochanteric fractures (IF) in the elderly are often complicated by osteoporosis and high rates of fixation failure. Current treatment options have limitations in providing both stable fixation and early mobilization in this fragile population. This study aimed to introduce and evaluate a novel approach, the periprosthetic femoral fracture treatment concept (PFFtc), as a surgical strategy to guide hip arthroplasty in elderly IF patients.

**Methods:**

A retrospective analysis was conducted on 209 elderly patients (mean age: 81.6 years; range: 70–93) with IF who underwent hip arthroplasty using the PFFtc protocol between March 2014 and August 2021, comprising 133 females and 76 males. All patients underwent treatment with the “PFFtc” and were subsequently followed up at intervals of 1 month, 3 months, 6 months, 1 year, 2 years, and annually thereafter. Clinical parameters such as ASA anesthesia grading, Visual Analogue Scale (VAS) scores, Harris Hip Scores (HHS), and Short‐Form 36 (SF‐36) outcomes were meticulously recorded. The subsidence of the femoral stem was assessed using Pelligrini's method, while mortality rates, postoperative complications, and patient's survival status post‐discharge were systematically documented. Multivariate logistic regression analysis was performed to identify independent risk factors for postoperative complications.

**Results:**

Over a mean follow‐up of 38.5 ± 6.0 months, prosthesis subsidence averaged 2.2 mm and stabilized. No deaths occurred within 30 days postoperatively. The 1‐ and 2‐year cumulative mortality rates were 4.3% and 11%, respectively. The most common complications included DVT and urinary tract infections. Logistic regression identified hypoproteinemia (OR = 2.38, *p* = 0.032) and heart disease (OR = 2.74, *p* = 0.012) as independent risk factors for postoperative complications. At final follow‐up, the mean VAS was 1.1 ± 1.0, HHS was 89.4 ± 3.9, PCS was 53.2 ± 8.5, and MCS was 50.5 ± 6.7. Among surviving patients, 63.0% lived independently at home.

**Conclusion:**

The PFFtc‐guided arthroplasty approach appears to be a safe and effective option for managing IF in elderly patients. It provides stable fixation and functional recovery of prostheses and muscles and offers a promising alternative to traditional fixation strategies.

## Graphical abstract

Anatomical images of the front (A) and back (B); the black lines indicated the muscles. One cable in “figure‐of‐eight” and another looped over the lesser trochanter were across the muscles to maintain reduction and resist traction.

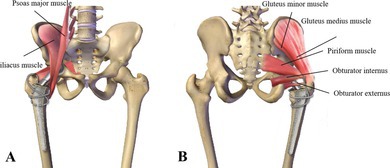

## 1 Introduction

Global projections estimate 21 million disabilities attributable to hip fractures in the next 40 years [1], imposing escalating strain on healthcare infrastructures and socioeconomic systems worldwide [2]. Intertrochanteric fractures (IF) constitute nearly half (about 45%–50%) of all geriatric hip fractures [3, 4, 5], necessitating urgent therapeutic optimization. Timely surgical intervention is paramount in minimizing complications, alleviating pain, restoring mobility, and enhancing the quality of life for patients in their advanced years [4, 6, 7, 8, 9].

Although open reduction and internal fixation (ORIF) is widely recognized as the standard approach for treating IF [4, 6, 10, 11], it is associated with a failure rate of 10%–25% in osteoporotic patients [10]. Hip arthroplasty has emerged as a proven and effective treatment modality for IF [5, 7, 12], with its adoption steadily increasing [13]. A meta‐analysis conducted by Jiang et al. [14] revealed that hip arthroplasty not only yields superior functional outcomes in the mid‐term but also significantly reduces the risk of reoperation compared to ORIF.

We posited that, following stem insertion during hip arthroplasty for IF, the situation could be analogously treated as a periprosthetic femoral fracture (PFF). This rationale led to the development of a novel treatment paradigm termed the “PFF treatment concept (PFFtc).” The purposes of this study were to: (i) apply the PFFtc principles to guide hip arthroplasty, including appropriate prosthesis selection and fixation strategy; (ii) achieve stable fixation of fracture fragments after anatomical or functional reduction; (iii) minimize postoperative complications while promoting early functional recovery in elderly patients with IFs.

## 2 Patients and Methods

This study was conducted with the explicit approval of the Hospital Ethics Committee from The First Affiliated Hospital of Zhejiang Chinese Medical University (No. 2016‐K‐143‐01), ensuring adherence to ethical standards and guidelines. Informed consent was obtained from all participants before their inclusion in the research. All patient data were anonymized before analysis to protect personal privacy. Identifiable information was removed, and data analysis was conducted in accordance with the Declaration of Helsinki.

### 2.1 Patients' Information and Clinical Assessment

Inclusion criteria: (1) Patients diagnosed with IF who underwent hip arthroplasty; (2) Age ≥ 70 years; (3) Availability of complete and comprehensive medical records; (4) Patients who were ambulatory before the injury.

Exclusion criteria: (1) Patients currently receiving hormone therapy or other medications known to exacerbate osteoporosis; (2) Cases involving pathological fractures; (3) Patients with a history of previous surgery on the ipsilateral proximal femur; (4) Presence of ipsilateral lower extremity injuries; (5) Patients who declined to comply with surgical procedures or postoperative management protocols; (6) Patients classified as American Society of Anesthesiologists (ASA) [15] anesthesia grade V or higher.

A total of 209 patients, comprising 133 females and 76 males, were enrolled in this study between March 2014 and August 2021. Detailed demographic information of the patients is presented in Table 1, while Table 2 outlines the underlying medical conditions. Preoperative radiographs were reviewed to assess the presence and severity of hip osteoarthritic changes using the Kellgren–Lawrence (KL) classification system [16]. The KL grading evaluates osteophyte formation, joint space narrowing, subchondral sclerosis, and bony deformity; KL ≤ 2 was regarded as non‐severe osteoarthritis, and ≥ 3 was classified into severe osteoarthritis. All included patients underwent hemiarthroplasty, with the use of a fully hydroxyapatite‐coated long stem during the surgical procedure.

**TABLE 1:** Basic patients' information.

Element	Number	Mean ± SD	Range
Patients	209		
Sex (female/male)	133/76		
Injured limb (left/right)	118/91		
Age at surgery (years old)		81.6 ± 5.6	70–93
BMI (kg/m^2^)		23.3 ± 3.5	16.8–30.4
Time from injury to surgery (h)		63.9 ± 33.3	15–164
T value		−3.5 ± 0.7	−2.5 to −5.4
ASA anesthesia grading			
Grade I	37		
Grade II	70		
Grade III	87		
Grade IV	15		
Kellgren–Lawrence grade			
Non‐serve osteoarthritis	29		
Serve osteoarthritis	180		

Abbreviations: BMI, body mass index; SD, standard deviation; T value, osteoporosis indicators, from routine lumbar vertebra bone density examination.

**TABLE 2:** Underlying diseases of patients.

Basic diseases	Total of number
Osteoporosis	209
Hypertension	119
Hypoalbuminemia (< 35 g/L)	108
Anemia	73
Mild	47
Moderate	24
Severe	2
Diabetes	75
Heart disease	
Coronary heart disease	44
Dilated cardiomyopathy	4
Valvular heart disease	14
Post pacemaker surgery	3
Lung disease	
Chronic obstructive pulmonary disease	37
Asthma	7
Pulmonary heart disease	5
Sequelae of cerebral infarction	13
Kidney disease	
Chronic kidney disease I	5
Chronic kidney disease II	12
Chronic kidney disease III	2
Malignant cancer	6
Pancreatic cancer	1
Lung cancer	2
Gastric cancer	2
Liver cancer	1

### 2.2 Preoperative Preparation

Preoperative management involved the meticulous optimization of pre‐existing medical conditions, including hypertension, hyperglycemia, and cardiovascular or pulmonary disorders, which could potentially compromise surgical outcomes. This process was conducted in close collaboration with an internist. Furthermore, upon admission, B‐ultrasonography of the deep veins in the lower extremities was systematically performed to exclude the presence of thrombosis.

### 2.3 PFFtc and Indications

The PFFtc was a surgical strategy that applied principles from the management of PFF to primary hip arthroplasty for IF. It emphasized achieving stable fixation of fracture fragments, restoring muscle attachment points, and enabling early mobilization through the use of long‐stem prostheses combined with cable‐based fixation techniques. In this study, PFFtc was indicated for elderly patients with IF, osteoporosis, comminuted fracture patterns involving the trochanteric region, or coexisting hip osteoarthritis, in whom ORIF was deemed unsuitable or at high risk of failure.

### 2.4 Surgical Technique

**FIGURE 1:**
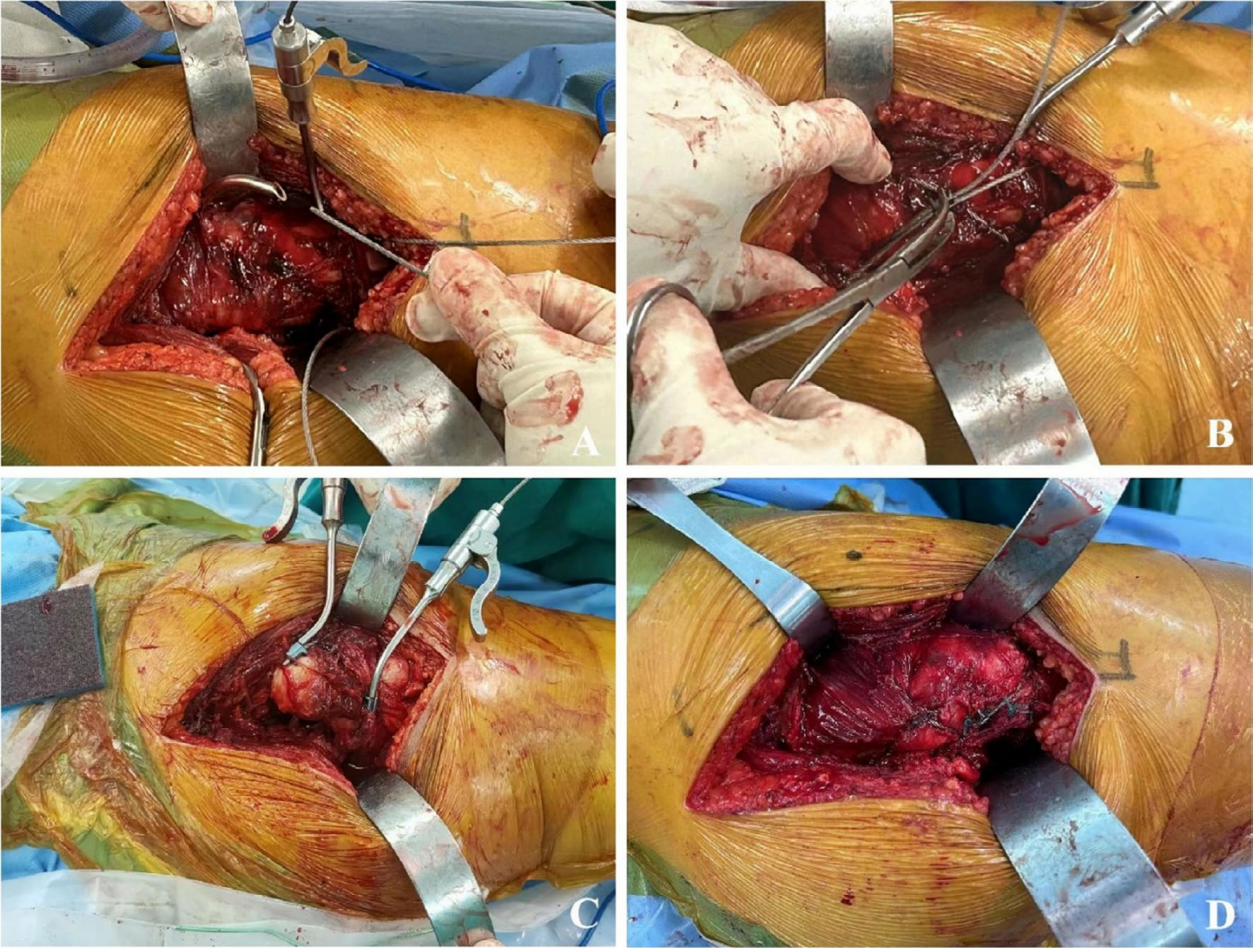
(A) A guidewire was passed through the cable at the muscle attachment site of the greater trochanter, while the cable loop was prebound below the lesser. (B) The cable was retrieved and positioned across the fracture line in “figure‐of‐eight,” fixation strategy details were in Figure 2. (C) Another cable was tied above the lesser trochanter, and both two cables were pre‐tightened. (D) After hip arthroplasty, the external rotator muscle group was sutured.

**FIGURE 2:**
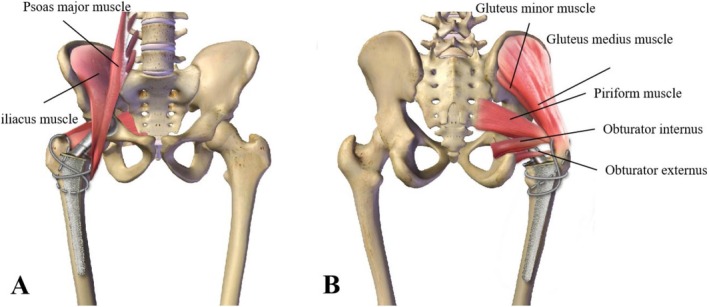
Anatomical images of the front (A) and back (B), the black lines indicated the muscles. One cable in “figure‐of‐eight” and another looped over lesser trochanter were across the muscles to maintain reduction and resist traction.

After satisfied anesthesia, the patient was placed in the lateral decubitus position. A nearly 10–15 cm posterolateral incision was made, the partial external rotator muscle group was severed, and then the joint capsule was cut. If the fracture was comminuted, two cables were used for pre‐binding (Figure 1A,B); then hip arthroplasty was performed. The binding method was as follows (Figure 2): one cable in “figure‐of‐eight” binding method was applied across the muscles, bundling one end below the lesser trochanter and the other end on the greater trochanter. Another cable was looped above the lesser trochanter for fixation. Femoral neck osteotomy was performed, then osteotomes of varying diameters were used to gradually expand the medullary canal and make a trial until an appropriately longer stem that fit the patient's conditions could be inserted. A fully hydroxyapatite‐coated long stem was selected; the length was determined intraoperatively based on the fracture extension, bone quality, and the need to bypass the fracture site to achieve distal fixation. Subsequently, appropriate hip joint abduction and adduction movements were performed to ensure stability without dislocation. The lengths of both lower limbs were compared. Tightened the pre‐binding cables (Figure 1C), and X‐rays were taken through the C‐arm machine. The joint capsule and external rotator muscle group (Figure 1D) were sutured, the incision was closed layer by layer, and the surgery was completed.

### 2.5 Postoperative Management

During the perioperative period, prophylactic antibiotics were administered within the first 24 h. Low‐molecular‐weight heparin was used to prevent thrombus formation.

Pain control and anti‐osteoporosis treatments were routinely applied.

A physiotherapist‐guided rehabilitation program was initiated, including passive quadriceps exercises and ankle pump movements.

Patients were encouraged to begin partial weight‐bearing with assistive devices on the first postoperative day, based on their individual condition and tolerance.

### 2.6 Follow‐Up

Follow‐up was conducted at 1 month, 3 months, 6 months, 1 year, 2 years, and then yearly thereafter until the last follow‐up. Radiological assessments, including anteroposterior and lateral radiographs of the hip joint, were meticulously analyzed to detect any subsidence in comparison to prior images, utilizing the method outlined by Pelligrini [17]. Complications such as nonunion, infection, prosthesis loosening, fractures, and dislocations were diligently documented, along with mortality data. In addition, readmissions related to the surgical procedure or other complications were recorded. Union time was defined as the period required for the formation of at least three cortical bone bridges across the fracture line [18].

Functional outcomes were comprehensively evaluated using the Visual Analogue Score (VAS) [19], Harris Hip Score (HHS) [20], and the Short‐Form 36 (SF‐36) [21] health status questionnaire, which included both the physical component score (PCS) and mental component score (MCS). All data were independently assessed by two attending physicians. The readmission status directly associated with the surgery, as well as the patients' overall living conditions post‐discharge, were also meticulously recorded.

### 2.7 Data Analysis

Measurement data such as surgical information and scores during the follow‐up were expressed as mean ± standard deviation (SD). Count data were expressed as numbers or percentages. The Shapiro–Wilk test was performed to assess the normality of continuous variables before selecting the appropriate statistical tests. Normally distributed variables were compared using the independent‐samples *t*‐test, while non‐normally distributed variables were analyzed using the Mann–Whitney *U* test.

Multivariate logistic regression analysis was conducted to identify independent risk factors associated with postoperative complications. Covariates included in the model were selected based on clinical relevance and previous literature, and included age group (≥ 80 vs. < 80 years), sex (male vs. female), body mass index (BMI ≥ 24 vs. < 24), KL grade (severe vs. non‐severe), and the presence of comorbidities such as hypertension, anemia, hypoproteinemia, diabetes, heart disease, lung disease, renal disease, and malignancy. Odds ratios (ORs) with 95% confidence intervals (CIs) and corresponding *p* values were reported. Probability values less than 0.05 were considered significant. Data were analyzed by using SPSS software (ver 22.0; SPSS Inc., Chicago, IL, USA).

Before data analysis, a sample size calculation was conducted based on published data. Using a 1‐year mortality rate of 11.8% reported by Grimsrud et al. [5], a 95% confidence level, and a margin of error of ±5%, the required minimum sample size was calculated to be approximately 160 patients. This study included 209 patients and confirmed the reliability of the results.

## 3 Results

### 3.1 Perioperative Outcomes

No fatalities occurred during the perioperative period. The mean operative duration was 78.0 ± 15.5 min (range: 52–136). Intraoperative blood loss averaged 177.2 ± 49.4 mL (range: 50–280), and the average hospital stay was 11.5 ± 3.8 days (range: 6–24). Fracture union was achieved in 15.3 ± 1.3 weeks (range: 12.8–18).

### 3.2 Perioperative Complications

**FIGURE 3:**
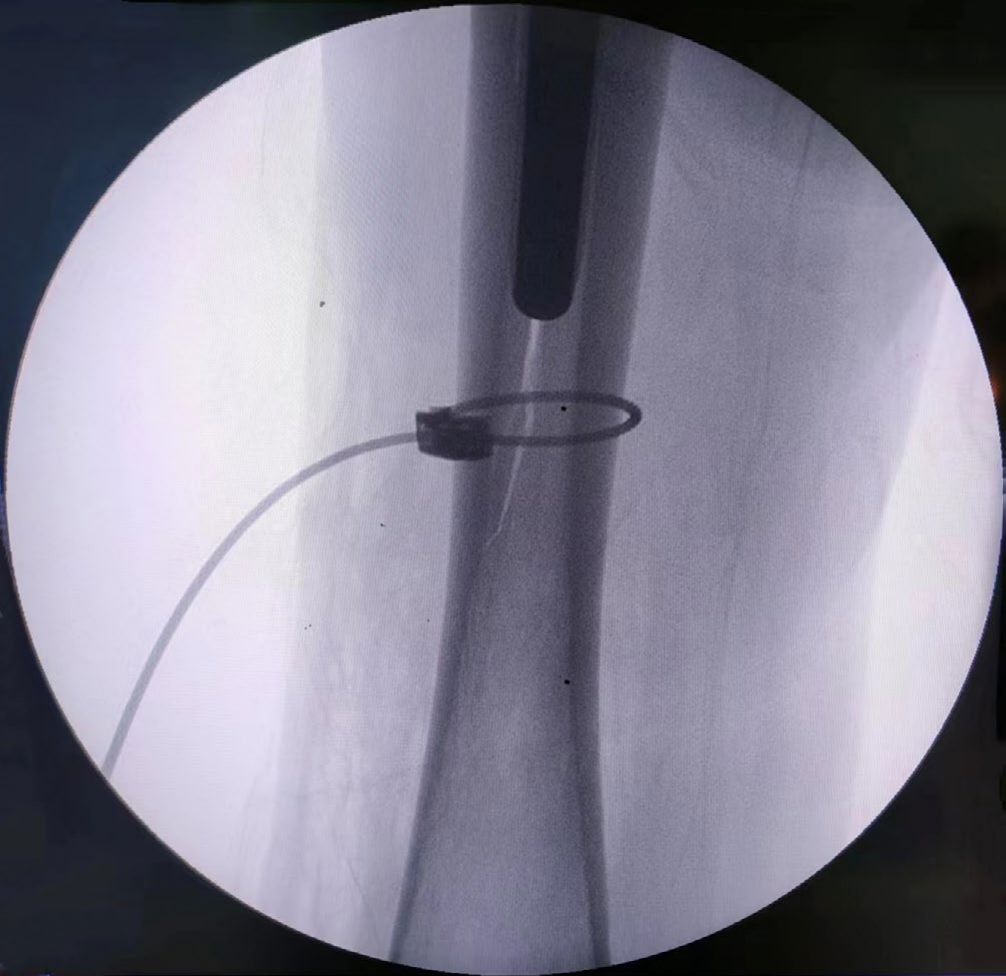
C‐arm image showed a distal periprosthetic femur fracture intraoperatively.

One patient sustained a distal femoral fracture due to improper femoral prosthesis insertion (Figure 3), which was managed intraoperatively with cable fixation. Deep vein thrombosis (DVT) was identified in 24 cases during hospitalization, involving the intermuscular vein (14 cases), popliteal vein (6 cases), and peroneal vein (4 cases). Of these, 7 were treated with inferior vena cava filter placement, while 17 received anticoagulant therapy. Postoperative urinary tract infections occurred in nine cases, all of which resolved with antibiotic treatment.

### 3.3 Follow‐Up Outcomes

Two patients who were lost to follow‐up (one was lost at 11.4‐month follow‐up and the other one was at 17.6 months) were excluded from long‐term mortality and functional analyses but were included in the perioperative complication analysis. At a mean follow‐up of 38.5 ± 6.0 months (range: 24–54.5), prosthesis subsidence was observed in 52 cases, averaging 2.2 mm (range: 1.1–3.0), and all cases stabilized by the final follow‐up. No PFFs, infections, or dislocations were reported. During the follow‐up period, 66 patients experienced accidental falls, resulting in lumbar vertebral fractures (3 cases), pelvic fracture (1 case), contralateral hip fractures (2 cases), and trochanteric displacement (1 case), yielding a fall rate of 31.6%. The remaining falls did not result in significant complications.

Continuous functional scores recorded during follow‐up are detailed in Table 3. Post‐discharge living arrangements were documented as follows: 116 of 184 patients (63.0%) resided in their own homes, 51 (27.7%) in nursing homes, and 17 (9.2%) were hospitalized for treatment of other comorbidities.

**TABLE 3:** The recording score during the follow‐up.

Score	Mean ± SD
HHS	
One month	49.6 ± 9.0
Three months	70.4 ± 7.9
Six months	81.2 ± 7.5
One year	86.5 ± 6.0
Two years	89.4 ± 4.6
Last follow‐up	89.5 ± 3.9
PCS	
One month	34.6 ± 6.3
Three months	44.8 ± 4.7
Six months	50.5 ± 5.2
One year	52.9 ± 6.6
Two years	53.9 ± 6.8
Last follow‐up	53.2 ± 8.5
MCS	
One month	27.1 ± 7.8
Three months	41.6 ± 6.3
Six months	48.4 ± 5.7
One year	51.1 ± 6.3
Two years	51.5 ± 7.3
Last follow‐up	50.5 ± 6.7
VAS	
One month	2.8 ± 0.8
Three months	1.9 ± 0.7
Six months	1.4 ± 0.9
One year	0.5 ± 0.7
Two years	0.7 ± 1.0
Last follow‐up	1.1 ± 1.0

Abbreviations: HHS, Harris hip score; MCS SF‐36, mental component; PCS SF‐36, physical component score; SD, standard deviation; VAS, Visual Analogue Scale.

### 3.4 Mortality

Among 209 patients, 15 succumbed to severe complications of COVID‐19, 5 to malignant tumors, 2 to heart disease, and 1 to advanced age, while 2 patients were lost to follow‐up. No deaths occurred within 30 days postoperatively, with 9 deaths within 1 year and 23 within 2 years. Among the 1‐year mortality cohort, 88.9% (8 patients) had an ASA score ≥ III, while 11.1% (1 patient) had an ASA score < III. In the 2‐year mortality cohort, 82.6% (19 patients) had an ASA score ≥ III, and 17.4% (4 patients) had an ASA score < III.

### 3.5 Multiple Regression Analysis

The results showed that hypoproteinemia (OR = 2.38, 95% CI: 1.08–3.47, *p* = 0.032) and heart disease (OR = 2.74, 95% CI: 1.25–4.12, *p* = 0.012) were significantly associated with an increased risk of postoperative complications (Table 4). Other variables, including age ≥ 80 years, sex, BMI ≥ 24, KL grade, hypertension, anemia, diabetes, lung disease, renal disease, and malignancy, were not statistically significant predictors (*p* > 0.05).

**TABLE 4:** Comparison of multivariate logistic regression models for predicting complications.

Basic diseases	Reference	OR	*p*
Age group (years old)			
≥ 80	< 80	1.15 (0.51–2.59)	0.737
Gender			
Male	Female	0.96 (0.43–2.15)	0.930
Injured limb			
Left	Right	1.17 (0.55–2.52)	0.682
BMI (kg/m^2^)			
≥ 24	< 24	0.76 (0.34–1.70)	0.506
Kellgren–Lawrence grade			
Serve osteoarthritis	Non‐serve osteoarthritis	0.54 (0.21–1.44)	0.219
Hypertension			
Yes	No	0.99 (0.46–2.13)	0.977
Anemia			
Yes	No	1.50 (0.69–3.25)	0.301
Hypoalbuminemia			
Yes	No	2.38 (1.08–3.47)	0.032*
Diabetes			
Yes	No	1.30 (0.60–2.80)	0.504
Heart disease			
Yes	No	2.74 (1.25–4.12)	0.012*
Lung disease			
Yes	No	1.22 (0.48–3.11)	0.680
Kidney disease			
Yes	No	2.02 (0.60–4.76)	0.253
Malignant cancer			
Yes	No	4.90 (0.76–30.41)	0.097

* *p* < 0.05, represents significant difference.

## 4 Discussion

### 4.1 Main Findings

This study evaluated the application of the PFFtc in elderly patients undergoing hip arthroplasty for IF. The main findings were: (i) PFFtc, involving the use of uncemented long‐stem prostheses and figure‐of‐eight cable fixation, achieved stable fixation without cable‐related complications; (ii) the approach yielded low perioperative mortality (0% at 30 days) and acceptable long‐term mortality rates (4.3% at 1 year, 11% at 2 years); (iii) hypoproteinemia and heart disease were identified as independent risk factors for postoperative complications; (iv) most patients achieved good functional outcomes and independent living at final follow‐up.

### 4.2 Hip Arthroplasty vs. ORIF for IF

Hip arthroplasty has become a widely adopted intervention for the management of IF [5, 7, 13, 22, 23, 24, 25, 26], particularly in elderly patients with osteoporosis, offering comparable functional outcomes to internal fixation without significantly increasing mortality or complication rates [27]. Although internal fixation methods such as the dynamic hip screw (DHS) have been considered a safer alternative to arthroplasty in elderly patients with multiple comorbidities, Zlámal et al. [28] reported satisfactory short‐term mobility and low complication rates in polymorbid patients with severe coxarthrosis; however, their study focused exclusively on stable IF. A recent systematic review by Steffann et al. [29] emphasized that arthroplasty may be particularly beneficial in patients over 70 years old with unstable fracture patterns, severe osteoporosis, and coexisting symptomatic hip osteoarthritis, as it can prevent the high rates of fixation failure and the need for complex revision surgeries. Patients with good pre‐fracture mobility may also benefit from early weight‐bearing after arthroplasty. In our study, all patients had osteoporosis, and most presented with KL grade ≥ 3 osteoarthritis, supporting the use of arthroplasty guided by the PFFtc strategy. However, fractures with severe comminution, cortical bone loss, or atypical patterns involving both the metaphysis and diaphysis pose additional challenges to fixation and healing, and outcomes in such cases may be unpredictable. Hip arthroplasty remains the standard salvage procedure following failed internal fixation [30, 31], further affirming its value in treating IF in the elderly.

### 4.3 Biomechanical Rationale and the PFFtc Approach

From both a biomechanical and clinical perspective, once a femoral stem was implanted during hip arthroplasty for an IF, the pattern of the surrounding bone fragments began to resemble those seen in PFFs. The Vancouver classification, which is widely used to categorize PFFs, offers a useful framework for understanding these cases [32] (Table 5). Fractures occurring near the femoral stem, such as those around the metaphysis or involving the greater or lesser trochanter, share similar characteristics with Vancouver types B1, B2, A_GT_, and A_LT_. Building on this similarity, the PFFtc proposed applying periprosthetic classification principles in primary arthroplasty for fracture cases, guiding surgical decisions and improving fixation and recovery in elderly patients.

**TABLE 5:** The Vancouver classification.

Type	Subtype	Characteristic
Type A		Fracture in intertrochanteric area
	A_LT_	Fracture at the greater trochanter
	A_GT_	Fracture at the lesser trochanter
Type B		Fracture around or just below the stem
	B1	Well‐fixed stem without loosening
	B2	Loose stem with good bone reserve
	B3	Loose stem with poor‐quality bone
Type C		Fracture distal to the stem

### 4.4 Fixation Strategy and Prosthesis Selection

The hip joint acts as the fulcrum for body weight and abductor muscles, with the dynamic balance between these forces being essential for pelvic alignment and normal gait [33]. The iliopsoas muscle, composed of the iliacus and psoas major, attaches to the lesser trochanter to stabilize the lumbar spine and hip joint while facilitating hip flexion and external rotation. The gluteus medius, piriformis, and gluteus minimus, attaching to the greater trochanter, are key to hip abduction, supported by the gluteus maximus in maintaining gait and joint stability. The recovery of muscle attachment points is crucial, especially the fixation of the greater trochanter, as failure may lead to a positive Trendelenburg sign and impair functional recovery [5, 15, 34]. Fortunately, until our last follow‐up, no cases of pelvic tilt or walking posture were observed.

**FIGURE 4:**
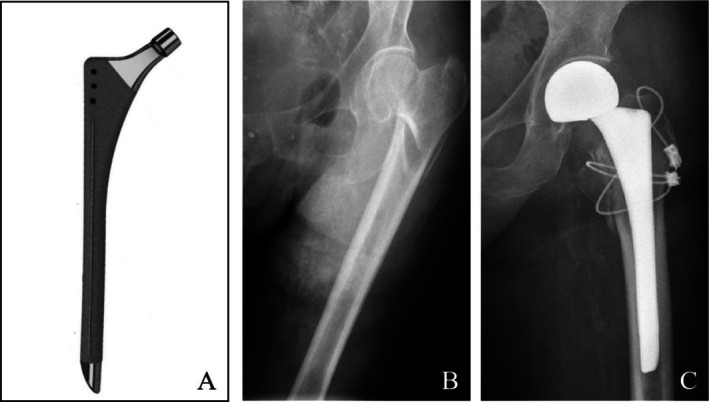
(A) The stem used in this study was a fully hydroxyapatite‐coated long stem. (B) Female, 93 years old, the anterior–posterior radiograph showed an intertrochanteric fracture. (C) The anterior–posterior radiograph after hip arthroplasty, the dissociative lesser trochanter and the greater trochanter were fixed by two cables.

To achieve stable fixation, two cables were used (Figures 2 and 4). One was placed proximally in a “figure‐of‐eight” pattern across the muscles, and the other passed through the muscles to secure the fragments above the lesser trochanter, providing basic anatomical reduction. Rather than directly fixing the trochanteric bone fragments, the cables counteracted muscle traction, preventing re‐displacement even without complete anatomical reduction [35]. This technique was supported by previous studies showing that figure‐of‐eight and cable‐based fixation can maintain trochanteric stability [5, 36].

Given the goal of stable fixation in PFFtc, prosthesis choice was critical. Cemented stems carry the risk of bone cement implantation syndrome (BCIS), a serious complication linked to increased mortality in elderly patients with cardiovascular disease or other comorbidities [37, 38]. Risk factors include ASA grade ≥ III, age > 75 years, and cardiopulmonary disease [39, 40]. BCIS presents as hypotension, arrhythmia, or hypoxia, and severe cases may involve pulmonary embolism from fat, marrow, or cement emboli due to elevated intramedullary pressure [41].

Short stems (< 120 mm) often provide only proximal stability and, in IF, may predispose to Vancouver B2 fractures due to stem loosening [42]. Bipolar long‐stem cementless prostheses, extending 4–6 cm beyond the fracture site, offer distal stability and reduce lateral stress [7, 11, 17, 31, 43, 44]. Compared with shorter stems, they minimize micromotion [45] and enhance distal femoral stability, as demonstrated by finite element analysis [46] and biomechanical studies [47]. The use of longer stems in this study likely contributed to the absence of type B2 fractures, as these stems provide enhanced distal stability. Instead, the fractures observed were primarily type B1 or type B1 combined with type A (A_GT_, A_LT_, or both). There was no statistically significant difference in the follow‐up scores with respect to fracture type, sex, or injured limb (*p* > 0.05).

### 4.5 Perioperative Management and Mortality Control

Belmont et al. [48] confirmed in a cohort of 17,640 patients that renal insufficiency and heart disease influence postoperative mortality after primary hip arthroplasty; other studies have indicated that underlying diseases and postoperative complications, rather than the surgical procedure itself, are the main determinants of mortality [49, 50]. Based on this, we strictly controlled underlying diseases preoperatively; in one case, surgery was delayed for nearly 164 h to achieve adequate control of hypertension, hyperglycemia, and severe underlying heart and lung disease. As a result, no patient died within 30 days, consistent with Grimsrud et al. [5]. The 1‐year mortality rate was 4.3%, lower than Grimsrud et al. (11.8%) [5] and Yu et al. (30%) [6], and the 2‐year mortality rate was 11%, also lower than Grimsrud et al. (24.7%) [5]. Therefore, controlling underlying diseases and preventing perioperative complications were essential for improving prognosis.

Although PFFtc provides favorable outcomes, its adoption may involve a learning curve, particularly in mastering the cable fixation technique and selecting appropriate prostheses. Adequate surgical training and experience are essential to ensure reproducible results.

### 4.6 Limitations and Prospect

First, due to the advanced age and limited life expectancy of some patients, conducting long‐term follow‐up was challenging. Second, patients with ASA grade V or above were excluded, and only uncemented long‐stem prostheses were used, which may have influenced the observed mortality rates. Third, as this was a retrospective, single‐arm study without a control group, the findings should be interpreted as preliminary. The study design was not intended to compare surgical techniques, such as PFFtc‐guided arthroplasty versus ORIF. Instead, it aimed to explore the feasibility and potential effectiveness of a treatment concept. The retrospective design inherently carries the risk of selection bias, highlighting the need for future prospective, multicenter studies to validate the findings.

## 5 Conclusions

PFFtc appears to be a promising approach for managing IF in elderly patients undergoing hip arthroplasty. Our findings suggest that the use of long‐stem uncemented prostheses combined with figure‐of‐eight cable fixation may provide stable fixation and satisfactory functional recovery. However, given the retrospective features, further prospective, multicenter, and controlled studies are needed to validate the clinical efficacy and safety of PFFtc.

## Author Contributions

**Wei‐Qiang Zhao:** designed the study, wrote the text, prepared and modified the figures and tables, approved the final version for submission. **Xu‐Song Li:** wrote the text, modified the text, modified the figures and tables, approved the final version for submission. **Ke‐Qin Yu:** wrote the text, collected the case, prepared the figures, and approved the final version for submission. **Rong‐Zhen Xie:** cases collected, analyzed the data, approved the final version for submission. **Jiang Hua:** cases collected, analyzed the data, approved the final version for submission. **Jie‐Feng Huang:** designed the study, modified the text, prepared and modified the figures, approved the final version for submission.

## Ethics Statement

Ethics committee approval was received for this study from the Institutional Review Board of The First Affiliated Hospital of Zhejiang Chinese Medical University (2016‐K‐143‐01).

## Consent

Informed consent was obtained from all participants.

## Conflicts of Interest

The authors declare no conflicts of interest.

## Data Availability

The data that support the findings of this study are available from the corresponding author upon reasonable request.
